# Circulating cell-free DNA and IL-10 from cerebrospinal fluids aid primary vitreoretinal lymphoma diagnosis

**DOI:** 10.3389/fonc.2022.955080

**Published:** 2022-08-18

**Authors:** Zhe Zhuang, Yan Zhang, Xiao Zhang, Meifen Zhang, Dongmei Zou, Li Zhang, Congwei Jia, Wei Zhang

**Affiliations:** ^1^ Department of Hematology, Peking Union Medical College Hospital, Chinese Academy of Medical Sciences & Peking Union Medical College, Beijing, China; ^2^ Department of Ophthalmology, Peking Union Medical College Hospital, Chinese Academy of Medical Sciences & Peking Union Medical College, Beijing, China; ^3^ Department of Clinical Laboratory, Peking Union Medical College Hospital, Chinese Academy of Medical Sciences & Peking Union Medical College, Beijing, China; ^4^ Department of Pathology, Peking Union Medical College Hospital, Chinese Academy of Medical Sciences & Peking Union Medical College, Beijing, China

**Keywords:** cerebrospinal fluid, circulating cell-free DNA, IL-10, *MYD88*, vitreoretinal lymphoma

## Abstract

Primary vitreoretinal lymphoma (PVRL) is a rare variant of primary central nervous system lymphoma (PCNSL) that presents diagnostic challenges. Here, we focused on circulating cell-free DNA (cfDNA) and interleukin-10 (IL-10) isolated from cerebrospinal fluid. Twenty-three VRL patients (17 PVRL, 2 PCNSL/O, and 4 relapsed VRL, from 10/2018 to 12/2021) and 8 uveitis patients were included in this study. CSF samples from 19 vitreoretinal lymphoma patients had sufficient cfDNA for next-generation sequencing. Of these patients, 73.7% (14/19) had at least one meaningful non-Hodgkin lymphoma-related mutation. The characteristic *MYD88* ^L265P^ mutation was detected in the CSF of 12 VRL patients, with a sensitivity, specificity, positive predictive value (PPV), and negative predictive value (NPV) of 63.2%, 100%, 100%, and 46.2%, respectively. No meaningful lymphoma related mutations were found in CSF samples from uveitis controls with typical intraocular lesions. Meanwhile, CSF IL-10 levels were elevated in 95.7% of the VRL patients, with a sensitivity, specificity, PPV, and NPV of 95.7%, 100%, 100% and 88.9%, respectively. Key somatic mutations like *MYD88* ^L265P^ and *CD79B* detected from CSF cfDNA and elevated CSF IL-10 levels can be promising adjuncts for primary vitreoretinal lymphoma diagnosis.

## Introduction

Primary vitreoretinal lymphoma (PVRL) is a rare extranodal non-Hodgkin lymphoma of the retina, vitreous, and optic nerve. Most PVRL patients are of the B-cell lineage; approximately 80% develop intracranial progression eventually, while 15%–20% of patients with primary central nervous system lymphoma (PCNSL) have intraocular involvement at diagnosis (1–4). Thus, PVRL is also considered a subset of PCNSL. Early diagnosis of vitreoretinal lymphoma benefits survival. PVRL often masquerades as chronic posterior uveitis, sometimes as retinitis in clinical manifestations, adding to the difficulties of diagnosis (5–7). Pathological diagnosis is the gold standard. However, the false negative rate of vitreous biopsy cytology or immune cytology is approximately 70% (8, 9). Flow cytometry of vitreous fluid increases the diagnostic sensitivity, up to 82% (10, 11).

In the recent years, numerous studies have focused on exploring potential ancillary techniques and biomarkers for primary vitreoretinal lymphoma (PVRL) diagnosis. The detection of PVRL characteristic gene mutations, including *MYD88* ^L265P^ and *CD79B*, and immunoglobulin gene rearrangement (4, 12–21) from aqueous humor or vitreous fluid has been considered effective diagnostic approaches. Meanwhile, elevate aqueous humor or vitreous fluid interleukin-10 (IL-10) levels and elevated IL-10/IL-6 ratios have been considered as useful biomarkers (22). Our previous studies also demonstrated the diagnostic and disease monitoring roles of cerebrospinal fluid (CSF) IL-10 levels in PCNSL patients (23, 24). Since PVRL is a special subset of PCNSL, we wonder whether CSF IL-10 levels are also elevated in primary vitreoretinal lymphomas.

Circulating cell-free DNA (cfDNA), double-strand DNA fragments (usually 130–180 base pairs) released from cells into surrounding blood or other body fluids by cellular breakdown or active secretion (25–28), has become a promising biomarker for solid tumors in the past decades (29). Numerous studies have demonstrated the diagnostic, monitoring, and prognostic role of plasma cfDNA in various types of solid tumors (30–38), and the recent use of plasma cfDNA in lymphoma genotyping and prognosis (39–42). For restricted-brain tumors, cerebrospinal fluid (CSF) cfDNA, rather than plasma cfDNA, provides a minimally invasive approach to detect tumor mutations and contribute to diagnosis (43–47).

Vitreous fluid is the key sample to PVRL diagnosis. However, the number of diagnostic tests is limited by the small sample size, while sample dilution adds to false-negative results. Considering the anatomic relationship between vitreoretinal and cerebrospinal fluid, and the role of CSF in PCNSL diagnosis, we conducted this study to evaluate the potential of baseline CSF cfDNA mutation profiles and IL-10 levels for VRL diagnosis and establish the foundation of serial CSF monitoring for early detection of intracranial progression in VRL patients.

## Methods

### Study design

Seventeen patients with PVRL, two patients with primary central nervous system lymphoma and intraocular involvement (PCNSL/O), and four patients with relapsed vitreoretinal lymphoma (RVRL) were included in this study as experimental group. All the patients were diagnosed in our center, from 10/2018 to 12/2021 and treated. The PVRL patients were enrolled from our two prospective single-center open-label phase II trials (NCT03746223 and NCT04899453), with the same diagnostic criteria as previously described (48). In brief, pathology is the gold standard for VRL diagnosis. Meanwhile, patients who fulfilled the following criteria of 1 + 2 and two of 3/4/5 were diagnosed with VRL, B-cell type: (1) clinical manifestations including typical vitreous opacities, subretinal lesions, or both; (2) aqueous humor or vitreous fluid IL-10/IL-6 >1; (3) vitreous pathology showing neoplastic lymphoma cells; (4) positive vitreous cell immunoglobulin gene rearrangement (IgH, Igκ, or Igλ); and (5) vitreous flow cytometry positive for lymphoma biomarkers. VRL patients with no evidence of CNS or systemic lymphoma were considered PVRL. Sometimes asymptomatic concurrent intracranial lesions were found in the routine head MRI; these VRL patients were considered PCNSL/O. RVRL patients were those who had previously been treated for systemic lymphoma (n=2) or vitreoretinal lymphoma (n=2), now experienced restricted intraocular relapse. Additionally, eight uveitis patients who presented with typical vitreous opacities and subretinal lesions were enrolled from 11/2019 to 12/2021 as suspected vitreoretinal lymphoma cases. Thorough exams ruled out the possibility of VRL; no malignancy presented on follow-ups (4.5–29 months).

This study conformed to the Declaration of Helsinki and was approved by the Institutional Ethics Committee of Peking Union Medical College Hospital. Written informed consent was obtained from each participant. Furthermore, patients with VRL received lumber puncture at baseline, before each chemotherapy and every 6 months during maintenance therapy to examine CSF and rule out CNS progression. Ten microliters of cerebrospinal fluid (CSF) and buccal mucosa were obtained from each patient prior to treatment for sequencing. Data on clinical characteristics were collected from electronic health records.

### DNA extraction, library preparation, and targeted DNA sequencing

Germline DNA was extracted from buccal mucosa using the DNeasy Tissue Kit (Qiagen, USA) according to the manufacturer’s guidelines. CSF was collected and processed within 4 h. CSF-derived circulating cell-free DNA (cfDNA) was extracted with the QIAamp Circulating Nucleic Acid Kit (Qiagen). Then, the fragment length and quantity of cfDNA were assessed with the Qubit Fluorometer, Qubit dsDNA BR Assay Kit (Invitrogen, USA), and Labchip GX Touch system (PerkinElmer, Shanghai, China). All libraries were hybridized to custom-designed biotinylated oligonucleotide probes (IDT, Coralville, IA, USA) covering 413 genes. DNA sequencing was performed using the GeneSeq-2000 (Geneplus-Suzhou, Suzhou, China) with a read length of PE100 and depth of 500–1,000× (49), and 90 genes related to lymphoma were used for subsequent analysis (**Supplementary Table S1**).

### Sequencing data processing and mutation calling

Terminal adaptor sequences and low-quality reads were removed separately from raw data of paired samples using NCrealSeq (version 1.2.0, Geneplus-Suzhou) and NCfilter (version 2.0.0, Geneplus-Suzhou). The Burrows–Wheeler Aligner (BWA, version 0.7.15-r1140) tool was used to align clean reads to the reference human genome (GRCh37). Duplicate reads of cancer sample derived from PCR amplification were marked using realSeq, which was designed to retain reads containing rare events by treating Unique Molecular Indexes, and the normal sample was marked using Picard tools (version 2.6.0).

Single nucleotide variants (SNVs) and Indels were detected by comparing tumor-normal pairs using TNscope (version 201808) and realDcaller (version 1.7.1, Geneplus-Suzhou), a software developed to review hotspot variants. The results of these analyses were merged using NChot (version 2.7.2, Geneplus-Suzhou) and then annotated to multiple public databases using NCanno (version 1.1.3, Geneplus-Suzhou). For somatic copy-number alteration, CNVKIT (version 0.9.2) (50) was performed, and the matched buccal mucosa samples served as matched controls. Significant copy number variations were calculated as the ratio of adjusted depth between case gDNA and control gDNA. NCSV (0.2.3, Geneplus-Suzhou) was used to identify split-read and discordant read-pair to identify SVs. All candidate variants were manually verified with the integrative genomics viewer browser (51).

### Cerebrospinal Fluid IL-10 Detection

One milliliter of fresh CSF samples was used to detect the levels of inflammation factor IL-10 as previously described (23, 24). In brief, CSF samples were centrifuged (10 min at 500×*g* at 18°C); then, the supernatants were collected, and the IL-10 levels were measured with an electrochemiluminescence immunoassay (ECLIA) analyzer following the manufacturer’s instructions (Siemens Immulite 1000 and IL-10 detection kits). The levels of detected CSF IL-10 range from 5 to 1,000 pg/ml.

### Statistical methods

RStudio was used to present the results of sequencing. As for validity measurement, sensitivity, specificity, positive predictive value (PPV), and negative predictive value (NPV) were calculated (52).

## Result

### CSF circulating cell-free DNA in patients with vitreoretinal lymphoma

**Figure 1:**
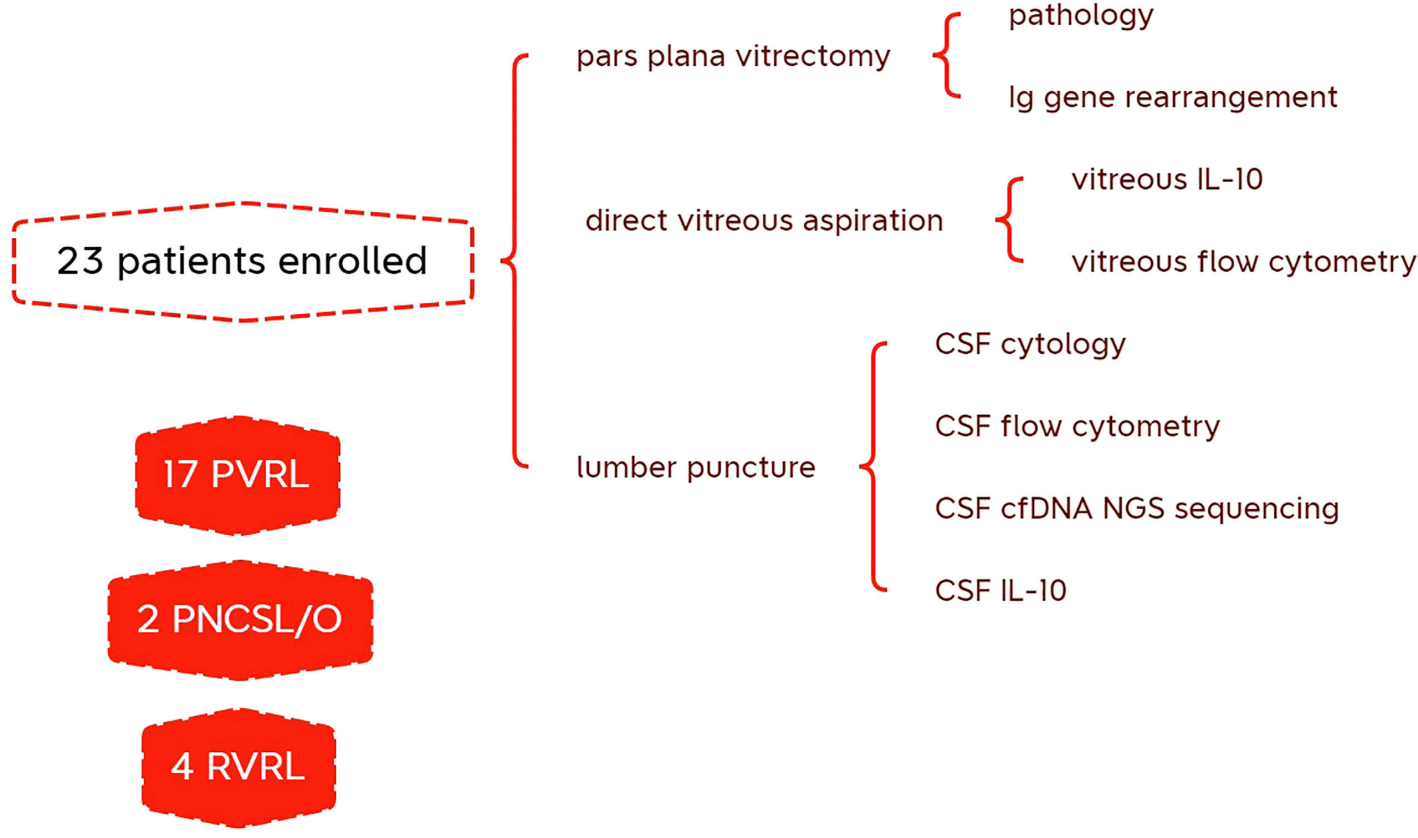
Schematic flowchart of diagnostic test performed on patients with suspected vitreoretinal lymphoma.

We collected CSF samples from 23 patients with B-cell vitreoretinal lymphoma, with or without CNS involvement and lymphoma history (**Table 1**). Specifically, 17 patients with primary vitreoretinal lymphoma, 2 VRL patients with concomitant CNS lymphoma, 2 patient whose vitreoretinal lymphoma had relapsed, and 2 patients whose previous systemic DLBCL had intraocular relapse were included in this study. Additionally, eight patients who presented with typical vitreous and subretinal lesions but without evidence of malignancy were included as controls, including idiopathic uveitis, cytomegalovirus retinitis, ocular sarcoidosis, and radiation retinopathy. All patients with suspected vitreoretinal lymphoma underwent several diagnostic tests, as shown in the schematic flowchart (**Figure 1**).

**Table 1:** Demographic and clinical information of vitreoretinal lymphoma patients.

ID	Diagnosis	Gender	Age	Intraocular lesions	Intraocular lesion location	Extraocular lesions at diagnosis	Previous disease
PVRL-01	PVRL	F	61	Bilateral	Vitreous + subretinal	NA	NA
PVRL-02	PVRL	M	48	Left	Vitreous + subretinal	NA	NA
PVRL-03	PVRL	M	52	Bilateral	Vitreous + subretinal	NA	NA
PVRL-04	PVRL	F	48	Bilateral	Vitreous	NA	NA
PVRL-05	PVRL	F	70	Bilateral	Vitreous	NA	NA
PVRL-06	PVRL	F	52	Right	Vitreous + subretinal	NA	NA
PVRL-07	PVRL	F	69	Bilateral	Vitreous + subretinal	NA	NA
PVRL-08	PVRL	F	44	Bilateral	Vitreous	NA	NA
PVRL-09	PVRL	F	54	Bilateral	Vitreous	NA	NA
PVRL-10	PVRL	M	62	Right	Vitreous + ciliary body	NA	NA
PVRL-11	PVRL	F	56	Bilateral	Vitreous + subretinal	NA	NA
PVRL-12	PVRL	F	69	Bilateral	Vitreous + subretinal	NA	NA
PVRL-13	PVRL	M	69	Bilateral	Vitreous + subretinal	NA	NA
PVRL-14	PVRL	M	39	Bilateral	Vitreous	NA	NA
PVRL-15	PVRL	F	41	Bilateral	Vitreous	NA	NA
PVRL-16	PVRL	F	51	Left	Vitreous + subretinal	NA	NA
PVRL-17	PVRL	F	49	Left	Vitreous + subretinal	NA	NA
PCNSL/O-21	PCNSL/O	M	61	Bilateral	Vitreous + subretinal	Right frontal lobe	NA
PCNSL/O-22	PCNSL/O	M	62	Bilateral	Vitreous + subretinal	Multiple intracranial lesions	NA
RVRL-31	RVRL	F	61	Right	Vitreous	NA	PVRL(Left)
RVRL-32	RVRL	F	50	Bilateral	Vitreous + subretinal	NA	DLBCL (breast, bone)
RVRL-33	RVRL	F	58	Bilateral	Vitreous	NA	DLBCL
RVRL-34	RVRL	F	52	Right	Vitreous + subretinal	NA	PVRL (Bilateral)
CONTROL-1	Idiopathic uveitis	M	64	Right	Vitreous + subretinal	NA	PCNSL
CONTROL-2	Idiopathic uveitis	F	58	Left	Vitreous + subretinal	NA	NA
CONTROL-3	Idiopathic uveitis	F	57	Right	Vitreous + subretinal	NA	NA
CONTROL-4	CMV retinitis	F	62	Bilateral	Vitreous + subretinal	NA	AITL
CONTROL-5	Ocular sarcoidosis	M	57	Bilateral	Vitreous + subretinal+ Ciliary body	NA	NA
CONTROL-6	Radiation retinopathy	F	47	Right	Vitreous + subretinal	NA	PCNSL
CONTROL-7	Idiopathic uveitis	F	58	Bilateral	Vitreous + subretinal	NA	NA
CONTROL-8	Idiopathic uveitis	M	61	Bilateral	Vitreous + subretinal	NA	NA

AITL, angioimmunoblastic T-cell lymphoma; CMV, cytomegalovirus; DLBCL, diffuse large B-cell lymphoma; NA, not applicable; PCNSL, primary central nervous system lymphoma; PCNSL/O, primary central nervous system lymphoma and intraocular involvement; PVRL, primary vitreoretinal lymphoma; RVRL, relapsed vitreoretinal lymphoma.

**Figure 2:**
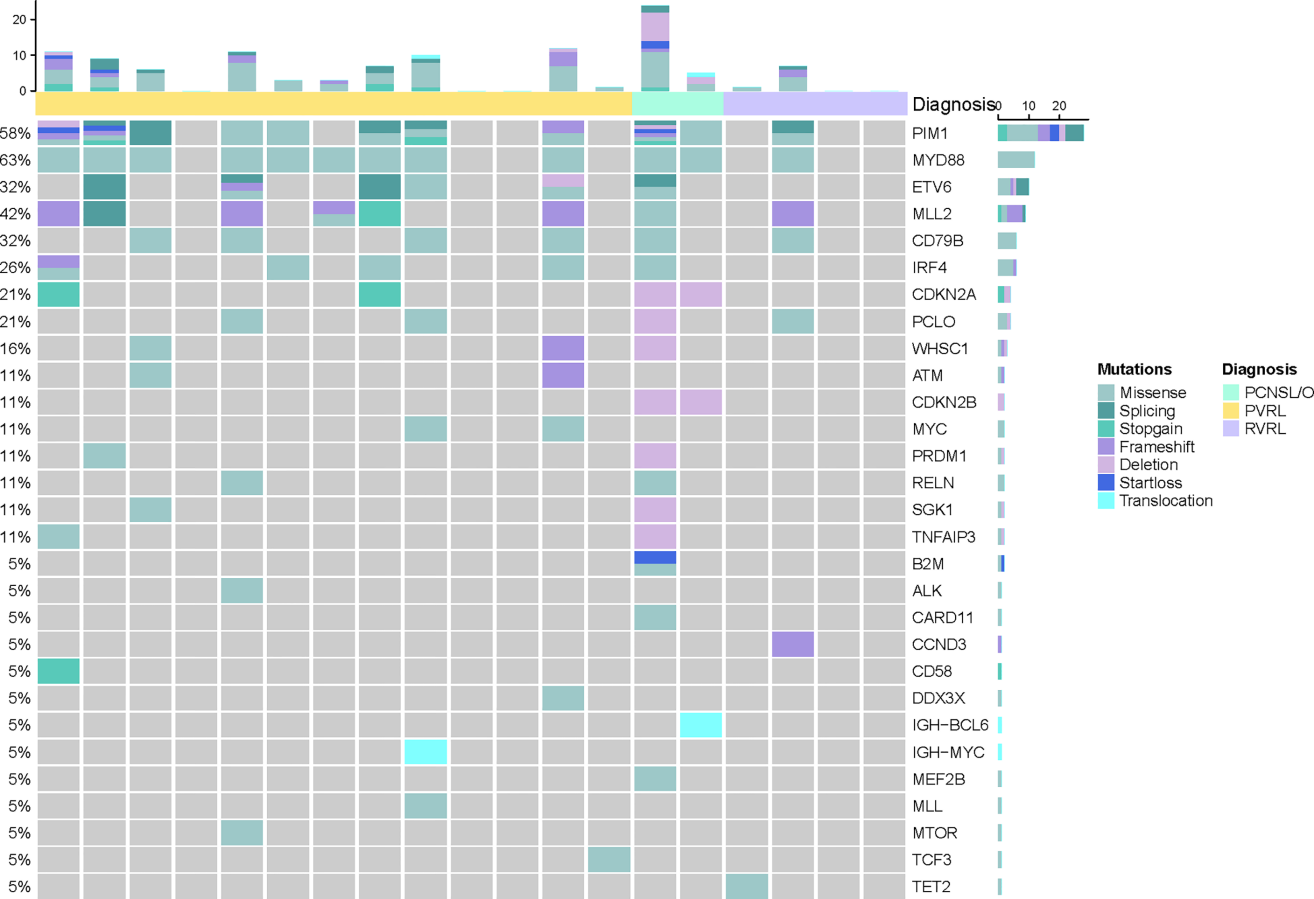
Baseline genomic characteristics of cerebrospinal cfDNA in vitreoretinal lymphoma patients.

**Figure 3:**
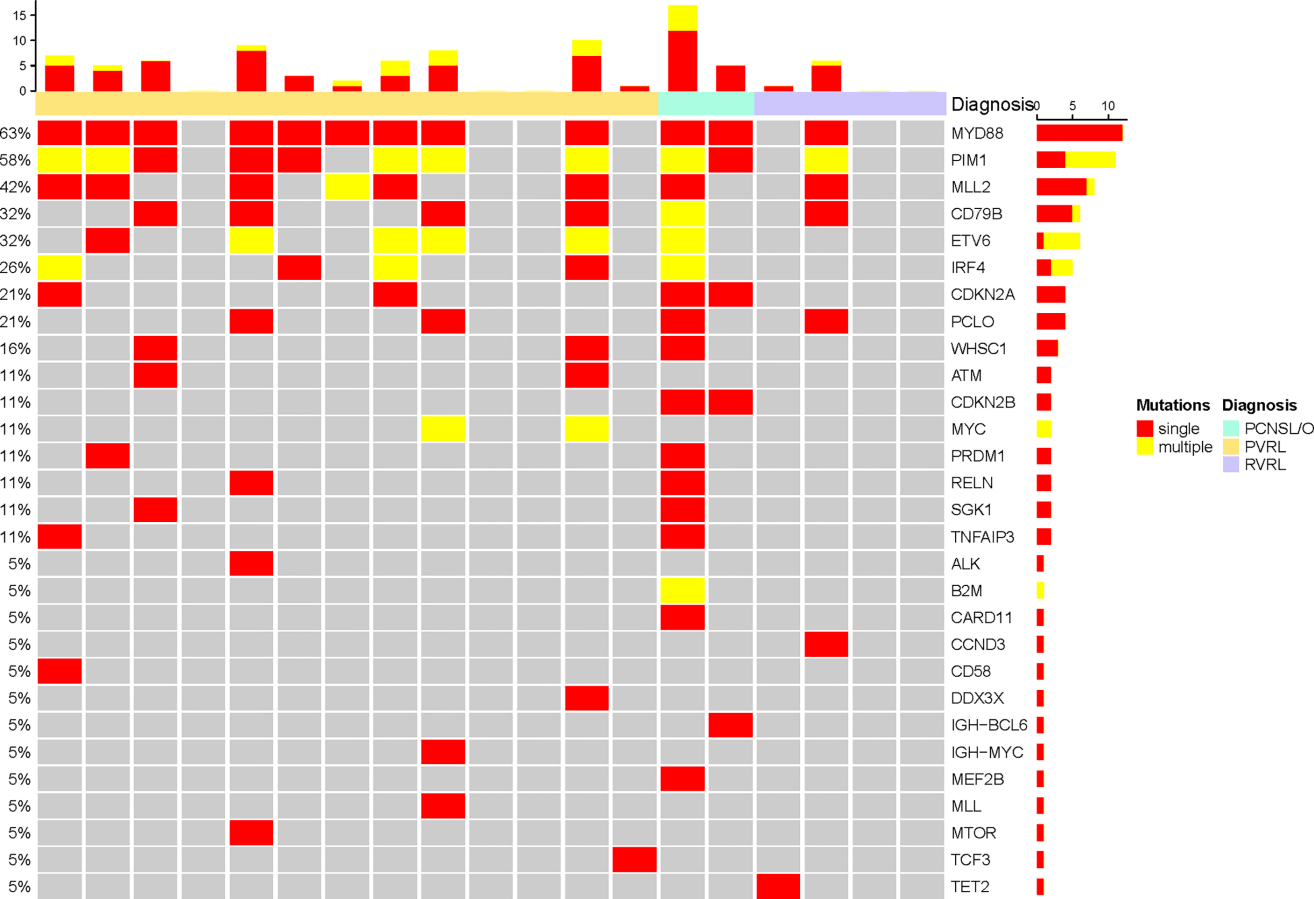
Single and multiple somatic mutations of cerebrospinal cfDNA in vitreoretinal lymphoma patients at baseline.

cfDNA was extracted from CSF; then, targeted deep sequencing of NHL-related genes was performed to identify somatic mutations (**Figures 2**, **3**). In four patients with primary vitreoretinal lymphoma, the amounts of extracted cfDNA (ranged from 0.1 to 0.6 ng) were not sufficient for cfDNA library construction, which failed to perform sequencing. Analysis of the cfDNA in the CSF of the remaining 19 vitreoretinal lymphoma patients revealed detectable mutations in 14 patients (**Figure 2**), at different variant allele frequencies (VAFs), ranging from 1.0% to 96.9%. In PVRL and PCNSL/O, 11/15 of the sequenced patients had *MYD88* ^L265P^ mutation, while 5 were with *MYD88* ^L265P^ and *CD79B* co-mutation. *PIM1* was the most frequently mutated gene. In the meantime, sequencing of non-lymphoma controls’ CSF cfDNA showed no mutation in five, insufficient cfDNA in two, and *DNMT3A* c.1851+1G>A mutation (VAF 0.9%) in one (CONTROL-5).

### CSF cfDNA is more sensitive than vitreous histology or flow cytometry in detecting vitreous–retina lesions at baseline

The detection of cfDNA in CSF was compared with the conventional methods of malignant cells identification at baseline (**Table 2**) and diagnostic validity was compared (**Table 3**). Although neoplastic lymphocytes were found in 22 out of the 23 VRL patients, most of the biopsy samples were not enough for immunohistochemical staining. Only four VRL patients were diagnosed with histology. Vitreous flow cytometry detected malignant B cells in 9 out of the 21 VRL patients tested, whereas vitreous cell immunoglobulin gene rearrangement detected 18 out of the 23. In our cohort, CSF cfDNA analysis revealed NHL-related gene mutations in 73.7% of the vitreoretinal lymphoma patients, with higher sensitivity than vitreous histology or flow cytometry (17.4% and 42.9%, respectively), slightly lower sensitivity than vitreous *Ig* gene rearrangement (78.3%). Notably, in the 14 cases that exhibited cfDNA mutation, 12 were with *MYD88* ^L265P^ mutation (overall sensitivity of 63.2%), and 6 were with *MYD88* ^L265P^ and *CD79B* co-mutation. Although in some cases lymphoma cells were not detected by cytology (PVRL-02, PVRL-05, PVRL-11, and PCNSL/O-21&22), the characteristic *MYD88* ^L265P^ mutation was detected in the CSF cfDNA (**Figure 2**). These findings suggest that sequencing CSF cfDNA can act as an adjunct approach to the diagnosis of VRL.

**Table 2:** Diagnostic tests of vitreoretinal lymphoma patients and uveitis controls.

ID	Histology	Vitreous cell Ig gene rearrangement	Vitreous FCM	Vitreous IL-10 (pg/ml)		Vitreous IL-10/IL6	CSF FCM	CSF cell number(×10^6^/L)	CSF WBC number(×10^6^/L)	CSF cfDNA*MYD88* ^L265P^	CSF IL-10 (pg/ml)

PVRL-01	Neoplastic lymphocytes	N	P	1,179.7		43.7	N	74	12	P	472
PVRL-02	Neoplastic lymphocytes	P	N	150.7		5.0	N	6	2	P	252
PVRL-03	Neoplastic lymphocytes	P	P	71.2		3.2	N	12	0	P	136.0
PVRL-04	NC	P	P	2,588.3		161.8	N	0	0	N	69.4
PVRL-05	Neoplastic lymphocytes	P	N	589.8		8.6	N	2	0	P	35.7
PVRL-06	Neoplastic lymphocytes	P	P	–		5.1	N	4	0	P	82.2
PVRL-07	Neoplastic lymphocytes	N	P	251.2		4.4	N	4811	10	P	108.0
PVRL-08	Neoplastic lymphocytes	P	P	282.3		5.8	N	6	2	IS	35.0
PVRL-09	DLBCL	P	ND	862		<1	N	6	2	IS	5.0
PVRL-10	Ciliary body–DLBCL	N	N	1,562.3		2.4	ND	0	0	IS	35.0
PVRL-11	Neoplastic lymphocytes	P	N	>10,000		>102	N	2	2	P	98.0
PVRL-12	DLBCL	N	N	–		12.5	N	14	8	P	62.7
PVRL-13	Neoplastic lymphocytes	P	P	1,089.7		90.8	N	26	2	N	83.0
PVRL-14	Neoplastic lymphocytes	P	N	592.2		4.25	N	2	0	N	57.3
PVRL-15	Neoplastic lymphocytes	P	ND	89.6		11.8	ND	4	4	IS	61.5
PVRL-16	Neoplastic lymphocytes	P	P	9.3		3	N	40	0	P	25.5
PVRL-17	Subretinal lesion–DLBCL	N	N	9,763.5		4.2	N	0	0	N	14.8
PCNSL/O-21	Neoplastic lymphocytes	P	N	2,154		25.8	N	6	2	P	388.0
PCNSL/O-22	Neoplastic lymphocytes	P	N	13,343.2		35.9	N	40	14	P	307.0
RVRL-31	Neoplastic lymphocytes	P	P	>20,000		>400	N	580	4	N	21.5
RVRL-32	Neoplastic lymphocytes	P	N	83.4		6.9	N	28	4	P	484.0
RVRL-33	Neoplastic lymphocytes	P	N	12,785.3		2.1	N	50	0	N	49.7
RVRL-34	Neoplastic lymphocytes	P	N	731.9		13.6	N	6	6	N	11.8
CONTROL-1	NC	N	N	–		1.8	N	0	0	N	7.4
CONTROL-2	Normal lymphocytes	N	N	1,203.8		19.3	N	4	2	N	6.8
CONTROL-3	NC	N	N	214.1		7.6	N	10	2	N	7.8
CONTROL-4	ND	ND	ND	19.6		<1	N	4	0	IS	5.0
CONTROL-5	Normal lymphocytes	N	N	46.7		<1	N	46	4	N	5.0
CONTROL-6	NC	N	N	–		<1	N	6	2	IS	5.6
CONTROL-7	NC	N	N	398.6		10.5	N	4	4	N	5.0
CONTROL-8	NC	N	N	170.4		24.3	N	4	4	N	5.0

The presented histology results were from vitrectomy, if not specified.

DLBCL, diffuse large B-cell lymphoma; IS, insufficient cfDNA for NGS, N, negative; NC, no cells detected; ND, not done; P, positive.

**Table 3:** Diagnostic validity of different diagnostic tests for vitreoretinal lymphoma.

	CSF *MYD88* ^L265P^	CSF IL-10	Vitreous cytology	Vitreous flow cytometry	Vitreous Ig gene rearrangement	Vitreous IL-10
Sensitivity	63.2%	95.7%	17.4%	42.9%	78.3%	95.7%
Specificity	100%	100%	100%	100%	100%	62.5%
Positive predictive value	100%	100%	100%	100%	100%	81.5%
Negative predictive value	46.2%	88.9%	32%	36.8%	58.3%	75%

### IL-10 levels are elevated in the CSF of vitreoretinal lymphoma patients

CSF IL-10 was previously demonstrated as a biomarker for the diagnosis and prognosis of primary central nervous system large B-cell lymphoma, with a cutoff value of 8.2 pg/ml (24). Here, we found that the levels of CSF IL-10 were also elevated in 22 out of the 23 vitreoretinal B-cell lymphoma patients, while the CSF IL-10 levels were within normal limits in the control uveitis group. Elevated CSF IL-10 levels had a sensitivity, specificity, PPV, and NPV of 95.7%, 100%, 100%, and 88.9% for the diagnosis of VRL, whereas those were 95.7%, 62.5%, 81.5%, and 75% for vitreous IL-10 levels. Our findings provide evidence that CSF IL-10 could be a good diagnostic marker for primary vitreoretinal lymphoma.

### Baseline CSF cfDNA levels or IL-10 level cannot predict treatment response

To determine whether baseline CSF biomarkers have a prognostic potential, we corrected cfDNA levels and their maximal somatic variant allelic frequency (maxVAF) with patients’ clinical outcome–progression-free survival time in the five patients treated with R2 (Rituximab combined with lenalidomide), as shown in **Table 4**. CSF cfDNA amount and maxVAF did not correlate with VRL PFS (p= 0.89, 0.55, respectively). Neither was the CSF IL-10 level (p=0.47).

**Table 4:** Baseline cfDNA and PVRL prognosis.

ID	Diagnosis	Treatment	Clinical outcomes	PFS (months)	Amount of cfDNA (ng)	maximal somatic variant allelic frequency
PVRL-01	PVRL	R2	PD (contralateral eye)	20.9	5.04	86.8%
PVRL-02	PVRL	R2	PD (Bilateral precentral gyrus)	9.2	2.10	94.8%
PVRL-03	PVRL	R2	PD	25.0	0.37	61.8%
PVRL-04	PVRL	R2	PD	11.0	0.60	0%
PVRL-05	PVRL	R2	PD (bilateral corpus callosum, cerebellum, lateral ventricle)	8.2	2.32	25.5%

## Discussion

The diagnosis of primary vitreoretinal lymphoma is still challenging. PVRLs usually present with bilateral blurry vision and floaters, anterior segment findings of keratic precipitates, and vitreous cellular infiltration of various severities (53). The clinical manifestations of primary vitreoretinal lymphoma are rather masquerading; patients are often misdiagnosed as intraocular inflammation or viral retinitis and wrongly treated (5–7). Cytological and immunohistochemical evidence of malignant lymphoma cells is the gold standard for diagnosis. However, the sensitivity of vitreous biopsy is disappointingly low, due to the lack of lymphoma cells in the vitreous specimen and necrosis during preparation (9). New diagnostic approaches like flow cytometry and molecular analysis of vitreous samples add to the diagnosis. Although Cani et al. (54) proposed with four patients that next-generation sequencing (NGS) test did not compromise the sample volume needed for other diagnostic tests, including cytology and flow cytology. From our experience, after cytology-based tests (cytology, immune cytology, and *Ig* rearrangement test) and flow cytology, the remaining vitreous samples could not provide enough DNA for NGS. Hence, we wondered whether CSF could be a substitute marker for PVRL diagnosis, since PVRL is a special subset of PCNSL and previous studies have demonstrated the diagnostic role of CSF cfDNA and elevated IL-10 levels (24, 55). Furthermore, serial CSF monitoring might be promising in the early detection of CNS progression in vitreoretinal lymphoma patients. To address the unmet needs of PVRL diagnosis, we conducted this study to analysis the diagnostic roles of CSF biomarkers, circulating cell-free DNA, and IL-10.

*MYD88* ^L265P^ is a unique non-synonymous point mutation in B-cell malignancies (56). Several studies demonstrated the presence of *MYD88* ^L265P^ mutation in the aqueous humor and vitreous fluid of vitreoretinal lymphoma patients. In different PVRL cohorts, the reported sensitivity of *MYD88* ^L265P^ mutation detection ranged from 25% to 88.9%, with direct Sanger sequencing of polymerase chain reaction (PCR), droplet digital PCR, or sequencing (12–19). The vitreous fluid samples showed higher positive rate than paired aqueous humor samples (14). We wondered whether *MYD88* ^L265P^ mutation also presented in the CSF of PVRL patients. In this study, we collected CSF samples from 31 patients with suspected VRL, then performed NGS. The final diagnosis was VRL in 23 patients. Despite the four samples without sufficient cfDNA for NGS, *MYD88* ^L265P^ mutation was confirmed in 12 of the remaining 19 VRL patients. Six patients were with *MYD88* ^L265P^ and *CD79B* co-mutation. Meanwhile, none of the uveitis controls contained the characteristic lymphoma mutations. The sensitivity, specificity, PPV, and NPV for using CSF *MYD88* ^L265P^ as VRL diagnostic marker were 63.2%, 100%, 100%, and 46.2%, respectively. Our findings suggest that key somatic mutations (i.e., *MYD88* ^L265P^) detected from CSF samples can be a promising additional approach for the accurate diagnosis of VRL. Notably, mutations without specific clinical meanings might be detected in non-lymphoma patients, like low frequency *DNMT3A* splicing mutation.

With CSF samples, we were able to overcome the difficulty of insufficient vitreous biopsy samples and picture the genomic features of vitreoretinal lymphomas. This can be a promising adjunct to vitreous fluid samples genomic analyses (19). Although there have been no standard treatment protocols for vitreoretinal lymphomas, the baseline mutation information presents targets for potential precision therapy, which might improve prognosis. Furthermore, we also need disease monitoring biomarkers for PVRL patients. For early detection of disease relapse and CNS progression, biomarkers like *MYD88* ^L265P^ or *CD79B* variant allele frequencies are promising. This study confirmed the presence of PVRL characteristic mutations in CSF, also established the foundation of assessing CSF samples to monitor disease progression. Furthermore, routine lumbar puncture for cerebrospinal fluid reduces the possible intraocular complications from aqueous puncture or vitreous aspiration.

IL-6 and IL-10 are the most extensively studied cytokines in vitreoretinal lymphomas; studies have demonstrated that elevated aqueous humor or vitreous fluid IL-10 and IL-10/IL-6 ratio can help distinguish vitreoretinal lymphomas from uveitis. However, there are also lymphoma cases with low IL-10 levels or non-lymphoma cases with elevated IL-10 levels (22, 57–59). In this study, we demonstrated that in patients with restricted intraocular lesions, 95.7% had elevated CSF IL-10 levels (ULN, 8.2 pg/ml), while the CSF IL-10 levels were within normal range in the controls. The sensitivity, specificity, PPV, and NPV were 95.7%, 100%, 100%, and 88.9%, respectively. The diagnostic accuracy of CSF IL-10 was slightly higher than vitreous fluid IL-10 (96.8% versus 80.6%). Meanwhile, parallel test of CSF *MYD88* ^L265P^ and CSF IL-10 levels showed a sensitivity of 98.4% and specificity of 100%. Furthermore, we have been detecting the IL-10 levels in serial CSF samples after treatment to assess whether IL-10 can be a potential disease monitoring biomarker.

A significant limit of our study is the small cohort size. Future studies with larger cohorts are needed. Furthermore, cfDNA extraction procedure still needs optimization to eliminate the effect of cfDNA degradation and increase the quantity of extracted cfDNA for sequencing. Nevertheless, we demonstrate that meaningful molecular data can be obtained from CSF cfDNA in PVRL patients. CSF *MYD88* ^L265P^ mutation and CSF IL-10 can be complementary approaches to the current diagnostic standard of PVRL. NGS of the CSF cfDNA also provides targets for precision therapy, including *MYD88*, *CD79B*, and *CDKN2A*. In the meantime, we have been collecting CSF samples of PVRL patients during therapy to investigate whether the mentioned biomarkers can monitor treatment response or indicate disease progression.

## Conclusions

Our study provides mutation landscape of vitreoretinal lymphomas with next-generation sequencing. *MYD88* ^L265P^ or *CD79B* mutation detected from CSF circulating cell-free DNA aids in primary vitreoretinal lymphoma diagnosis. Patient-specific genomic alterations are also pictured, which provide therapeutic targets for personalized medicine. Furthermore, IL-10 levels are also elevated in the CSF of VRL patients, with higher specificity than vitreous fluid IL-10.

## Data availability statement

The original contributions presented in the study are publicly available. This data can be found here: Link - https://ngdc.cncb.ac.cn/gsa-human/browse; Accession - HRA002732/HRA002732.

## Ethics statement

The studies involving human participants were reviewed and approved by the Institutional Ethics Committee of Peking Union Medical College Hospital. The patients/participants provided their written informed consent to participate in this study.

## Author contributions

ZZ, YZ, XZ, MFZ, and WZ designed the experiment. YZ, XZ, MFZ, and WZ enrolled participants and treated the enrolled patients. ZZ and DMZ collected patient samples and data. ZZ, DMZ, and LZ conducted the experiments. CWJ double checked all the histology samples. ZZ wrote the first draft manuscript. All authors edited and approved the manuscript.

## Funding

This study was funded by the CAMS Innovation Fund of Medical Sciences (CIFMS) 2019-I2M-2-009, the CAMS Innovation Fund for Medical Sciences (CIFMS) [2021-I2M-C&T-B-005], and Natural Science Foundation of Beijing Municipality 7202154.

## Acknowledgments

We thank all the patients for their consent in participating in the study and sharing their medical records. We thank the medical staff and physicians who participated in this study. We are also grateful to Dr. Nanzhao for her help in statistical analysis.

## Conflict of interest

The authors declare that the research was conducted in the absence of any commercial or financial relationships that could be construed as a potential conflict of interest.

## Publisher’s note

All claims expressed in this article are solely those of the authors and do not necessarily represent those of their affiliated organizations, or those of the publisher, the editors and the reviewers. Any product that may be evaluated in this article, or claim that may be made by its manufacturer, is not guaranteed or endorsed by the publisher.
