# Corrigendum: Circulating cell-free DNA and IL-10 from cerebrospinal fluids aid primary vitreoretinal lymphoma diagnosis

**DOI:** 10.3389/fonc.2022.1104236

**Published:** 2023-01-27

**Authors:** Zhe Zhuang, Yan Zhang, Xiao Zhang, Meifen Zhang, Dongmei Zou, Li Zhang, Congwei Jia, Wei Zhang

**Affiliations:** ^1^Department of Hematology, Peking Union Medical College Hospital, Chinese Academy of Medical Sciences & Peking Union Medical College, Beijing, China; ^2^Department of Ophthalmology, Peking Union Medical College Hospital, Chinese Academy of Medical Sciences & Peking Union Medical College, Beijing, China; ^3^Department of Clinical Laboratory, Peking Union Medical College Hospital, Chinese Academy of Medical Sciences & Peking Union Medical College, Beijing, China; ^4^Department of Pathology, Peking Union Medical College Hospital, Chinese Academy of Medical Sciences & Peking Union Medical College, Beijing, China

**Keywords:** cerebrospinal fluid, circulating cell-free DNA, IL-10, MYD88, vitreoretinal lymphoma

In the published article, there was an error in the Funding statement. The funding statement for the CAMS Innovation Fund for Medical Sciences was displayed as “special project of clinical and translational medicine research of CAMS 2021-I2M-C&T-B-005”.

The correct Funding statement appears below.

