# Supplementary material for: Circulating cell-free DNA and IL-10 from cerebrospinal fluids aid primary vitreoretinal lymphoma diagnosis
Source: Front Oncol. 2022 Aug 18;12:955080. doi: 10.3389/fonc.2022.955080 (PMC9434796; doi:10.3389/fonc.2022.955080)
Supplement: Supplementary file 1 [file Table_1.docx]

Supplementary Material

# Supplementary Table

**Table S1. Genes included in the panel**

| **90 genes** | | | | | |
| --- | --- | --- | --- | --- | --- |
| ALK | ARID1A | ARID1B | ATM | B2M | BCL2 |
| BCL6 | BCOR | BIRC3 | BRAF | BTK | CARD11 |
| CCND1 | CCND2 | CCND3 | CD274(PDL1) | CD28 | CD58 |
| CD79A | CD79B | CDKN2A | CDKN2B | CREBBP | CXCR4 |
| CXCR5 | DDX3X | DNMT3A | EP300 | EPHA7 | ERBB4 |
| ETV6 | EZH2 | FAS | FBXW7 | FGFR1 | FOXO1 |
| GATA3 | GNA13 | ID3 | IDH2 | IKZF1 | IRF4 |
| IRF8 | ITK | JAK1 | JAK2 | JAK3 | KIT |
| KLHL6 | KMT2A(MLL) | KMT2D(MLL2) | KRAS | MAP2K1 | EF2B |
| MTOR | MYC | MYD88 | NOTCH1 | NOTCH2 | AX5 |
| PCLO | PDCD1LG2(PDL 2) | PDGFRB | PHF6 | PIK3CA | IM1 |
| PLCG2 | PRDM1 | PTEN | RB1 | RELN | HOA |
| SETD2 | SF3B1 | SGK1 | SMARCA4 | SOCS1 | TAT3 |
| STAT5B | STAT6 | SYK | TCF3 | TET2 | NFAIP3 |
| TNFRSF14 | TP53 | TP63 | WHSC1 | WT1 | PO1 |
